# Supramolecular latching system based on ultrastable synthetic binding pairs as versatile tools for protein imaging

**DOI:** 10.1038/s41467-018-04161-4

**Published:** 2018-04-27

**Authors:** Kyung Lock Kim, Gihyun Sung, Jaehwan Sim, James Murray, Meng Li, Ara Lee, Annadka Shrinidhi, Kyeng Min Park, Kimoon Kim

**Affiliations:** 1 Center for Self-assembly and Complexity (CSC), Institute for Basic Science (IBS), Pohang, 37673 Republic of Korea; 2 Division of Advanced Materials Science, Pohang University of Science and Technology (POSTECH), Pohang, 37673 Republic of Korea; 3 School of Interdisciplinary Bioscience and Bioengineering, Pohang University of Science and Technology (POSTECH), Pohang, 37673 Republic of Korea; 4 Department of Nanomaterials and Engineering, University of Science and Technology (UST), Daejeon, 34113 Republic of Korea; 5 Department of Chemistry, Pohang University of Science and Technology (POSTECH), Pohang, 37673 Republic of Korea

## Abstract

Here we report ultrastable synthetic binding pairs between cucurbit[7]uril (CB[7]) and adamantyl- (AdA) or ferrocenyl-ammonium (FcA) as a supramolecular latching system for protein imaging, overcoming the limitations of protein-based binding pairs. Cyanine 3-conjugated CB[7] (Cy3-CB[7]) can visualize AdA- or FcA-labeled proteins to provide clear fluorescence images for accurate and precise analysis of proteins. Furthermore, controllability of the system is demonstrated by treating with a stronger competitor guest. At low temperature, this allows us to selectively detach Cy3-CB[7] from guest-labeled proteins on the cell surface, while leaving Cy3-CB[7] latched to the cytosolic proteins for spatially conditional visualization of target proteins. This work represents a non-protein-based bioimaging tool which has inherent advantages over the widely used protein-based techniques, thereby demonstrating the great potential of this synthetic system.

## Abstract (web summary)

Although protein-ligand pairs are useful tools for bioimaging, they are susceptible to enzymatic degradation and interference from endogenous species. Here, the authors show that a synthetic and bioorthogonal cucurbit[7]uril-guest binding pair can be used to visualize proteins in cells, overcoming limitations of protein-based platforms.

## Introduction

The streptavidin–biotin (SA–BT) pair is a naturally occurring protein–ligand binding pair with high binding affinity (*K* ~ 10^13^ M^−1^) in physiological conditions^1^. The high binding affinity has allowed SA–BT to be used as a non-covalent tool for a wide variety of bioapplications such as immobilization and purification of biomolecules, biosensing, and bioimaging^2–4^. Nevertheless, since SA–BT is a natural protein-based binding pair, it has unavoidable limitations—especially in a cellular environment such as being susceptible to enzymatic degredation^5^. In addition, its large size (52 kDa) hampers precise and accurate molecular-level imaging^6^, cell permeability and the strong interaction between SA and BT is practically irreversible, unless SA is denatured using harsh conditions, which are undesirable for protein analysis, especially for imaging. Furthermore, endogenous BT blocks the binding sites of SA^7,8^ and endogenously biotinylated proteins may generate false positives^9–11^. Additional treatments with commercial kits with skillful handling can help reduce the interference from endogenously biotinylated species. However, unaddressable issues remain in the use of the natural binding pair system for cellular bio-imaging. Therefore, a small molecule-based artificial binding pair system with ultrahigh binding affinity is urgently required.

Cucurbit[*n*]urils (CB[*n*], *n* = 5–8, 10, and 14), a family of pumpkin-shaped synthetic host molecules, have unique molecular recognition properties^12,13^. Especially, CB[7] forms ultrastable and highly specific host–guest complexes with its guests such as ferrocenemethyl- (FcA), adamantyl- (AdA), and diamantyl ammonium derivatives. The binding affinities (*K* ~ 10^12^–10^17^ M^−1^)^14–21^ are remarkably high, and comparable to or even higher than that of SA–BT. The strong binding of CB[7] with its guest, which is easily and selectively attached, is reminiscent of a mechanical latch^22^. The CB[7]-based ultrastable binding pairs have been utilized for various applications such as biosensors^23^, under-water adhesives^24^, protein fishing^25^, guest-regulated catalysis^26^, guest-responsive activation of therapeutic effects^27^, and single vesicle contents mixing assays^28^. However, the ultrastable host–guest interaction between CB[7] and AdA (or other tight binders) has not been exploited for cellular imaging, despite its own unique features that make it useful for cell biology including: (1) a stable chemical structure that is robust in a cellular environment and not affected by enzymatic degradation; (2) their bio-orthogonality in binding to biomolecules; (3) their small size compared to other protein-based binding pairs such as SA–BT enabling for efficient cellular uptake; (4) controllable binding affinity by treatment with competing guests allowing for selective dissociation of the host–guest assembly, on demand; and (5) scalability using known chemical synthetic methods for cost-effective and convenient uses. Recently, Urbach and Isaacs reported the synthesis of tetramethylrhodamine-conjugated CB[7] (dye-CB[7]) and its intracellular uptake into cells^29^. We have also reported the synthesis and use of a dye-CB[7], namely cyanine 3-conjugated CB[7] (Cy3-CB[7]) for in vitro imaging purposes^28,30^. Although both of them showed the great potential of CB[7]-dye conjugates as imaging probes, their ability to form ultrastable and controllable host–guest interactions has not been exploited for imaging of cellular biomolecules, structures, or processes.

**Fig. 1:**
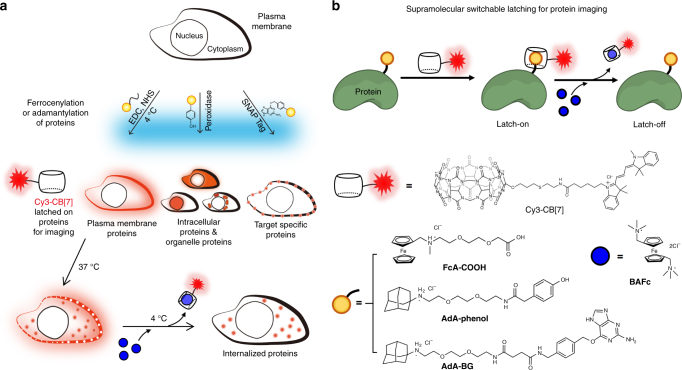
Schematic illustration of protein imaging. **a** Universal and selective imaging of cellular proteins using a switchable supramolecular latching system based on the ultrastable host–guest interactions between Cy3-CB[7] and AdA (or FcA); **b** CB[7]-based ultrastable binding pairs used in this study

Herein, we report the versatile use of Cy3-CB[7]–guest pairs as a bioimaging tool that can be used to specifically visualize proteins on the surfaces of cells and animals and in specific cellular compartments. We achieved this by employing a variety of different conjugation techniques to label cellular proteins of interest with AdA (or FcA) which then serve to localize Cy3-CB[7] at the cellular region of interest. In addition, using the reversible binding between Cy3-CB[7] and FcA (or AdA) labeled proteins, by treatment with a strong competing guest, the proteins that were internalized into cells were selectively visualized, by unlatching the Cy3-CB[7] from the cell surface proteins. (Fig. 1). This work represents the first example of a non-protein-based bioimaging platform which gives it inherent advantages over currently and widely used protein-based methods, such as resistance to proteases, a bioorthogonal binding mechanism, a small size and, easily controllable binding affinity. The aforementioned merits of the CB[7]–guest pairs are retained in cellular conditions; the most important of these being that the high-affinity host–guest binding is retained, which enables a ‘supramolecular latch-on’ between the labeled proteins and Cy3-CB[7]. Interestingly, the host–guest complex can be dissociated (the latch-off state) by guest exchange with a strong competitor, such as 1,1′-bis(trimethylammoniomethyl)ferrocene (BAFc), thereby providing selective conditional visualization of target proteins. Considering the synthetic nature, their bioorthogonality in binding and conditionally switchable visualization of cellular proteins of interest, we believe that the Cy3-CB[7]-guest pairs represent a general, non-protein-based and efficient cellular bioimaging tool for almost any target proteins which are labeled with AdA (or FcA).

## Results

### Ultrastable synthetic binding pair as a protein imaging tool

To demonstrate the versatility of our approach, we targeted several proteins in a variety of different locations, namely the plasma membrane, various intracellular compartments (the cytoplasm, nucleus, and mitochondria), and finally the surface of a live animal, *Caenorhabditis elegans*. Prior to imaging with Cy3-CB[7], using the concept of supramolecular latching, the proteins of interest were targeted by labeling with AdA (or FcA), a process we term adamantylation (or ferrocenylation), using various chemical and biological approaches as follows: (1) ferrocenylation of plasma membrane proteins using EDC (1-ethyl-3-(3-dimethylaminopropyl)-carbodiimide hydrochloride) coupling; (2) adamantylation of proteins in specific organelles using proximity-dependent enzyme-assisted labeling using horseradish peroxidases (HRPs) and an engineered ascorbate peroxidase (APEX); and (3) adamantylation of specific target proteins through the use of a self-labeling proteins tag, namely SNAP-tag^®^ (SNAP-tag) which can be expressed as a fusion with the target protein. To demonstrate the extra control that our system provides, conditional visualization of proteins was performed by exploiting the controllable binding of the host–guest complex. Cells were treated with a strong competing guest (BAFc) at a low temperature (4 °C), at which BAFc does not internalize, so only the Cy3-CB[7] on the cell surface was removed since it forms a more stable complex with BAFc (taking the supramolecular latch-off), while keeping Cy3-CB[7] inside the cells latched to the proteins.

**Fig. 2:**
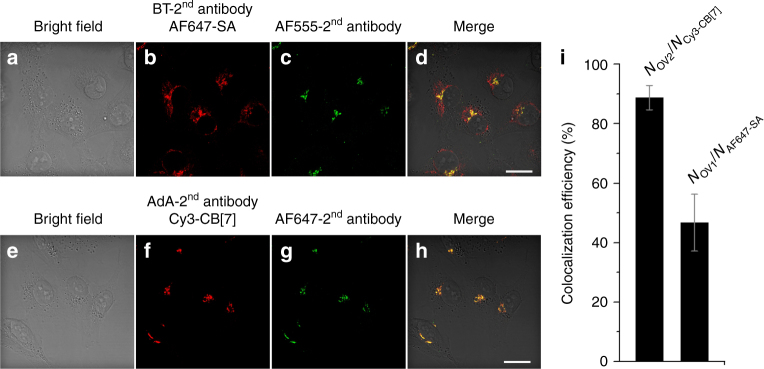
Visualization of Golgi using SA–BT and CB[7]-AdA. Confocal laser scanning microscopy images of Golgin97 monoclonal antibody treated MCF-7 cells **a**–**d** after treatment of BT-labeled secondary antibody with AF647-SA and AF555-secondary antibody, and **e**-**h** after treatment of AdA-labeled secondary antibody with Cy3-CB[7] and AF647-secondary antibody, scale bar = 20 μm. **i** A bar graph of colocalization efficiency of fluorescence signals between AF647-SA and AF555-secondary antibody, and Cy3-CB[7] and AF647-secondary antibody. The bar represents mean ± s.d. (see Methods for calculation of the colocalization efficiency and Supplementary Figure 5 for magnification of cells in Fig. 2.)

We first compared the imaging ability of Cy3-CB[7] to that of Alexa Fluor (AF) 647 (AF647)-SA to validate the ultrastable binding pairs as a protein imaging tool. As a test system, we decided to image an organelle, namely the Golgi. We used a Golgi-specific primary antibody (Golgin97 monoclonal antibody, CDF4) which selectively interacts with a *trans*-Golgi network protein, Golgin97. The primary antibody was recognized by AdA or BT-conjugated secondary antibody (see Supplementary Information for synthesis), which was later visualized by treating with Cy3-CB[7] or AF647-SA, respectively, under a confocal laser scanning microscope (Fig. 2). For a direct comparison of imaging ability for target proteins in the cells, AF647- and AF555-conjugated secondary antibodies were co-administered alongside Cy3-CB[7] and AF647-SA, respectively. The cells treated with the BT-secondary antibody showed more spread-out fluorescence signals from AF647-SA than that of the AF555-secondray antibody (Fig. 2d, Supplementary Figure 5A and C). This result indicated that AF647-SA visualized not only target proteins in Golgi but also off-target proteins in cytosol. This observation of unintended fluorescence signals over the whole cytoplasm, even after extensive washing, confirmed that the cells contained a non-ignorable amount of endogenously biotinylated proteins. However, in the case of the cells treated with AdA-secondary antibody, the fluorescence signals of Cy3-CB[7] was well-merged with that of AF647-secondray antibody (Fig. 2h, Supplementary Figure 5B and D), in contrast to that of AF647-SA with AF555-secondary antibody. We calculated the colocalization efficiency (Fig. 2i) of the fluorescence signals from AF647-SA and AF555-secondary antibody (49 ± 10%), and Cy3-CB[7] and AF647-secondray antibody (89 ± 4%) (see Methods for calculation); this quantitative analysis strongly suggested that the synthetic host–guest pair system provides more accurate and precise imaging of target proteins.

### Visualization of FcA-labeled plasma membrane proteins

**Fig. 3:**
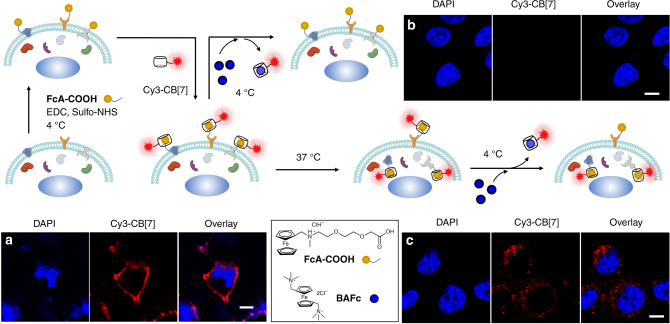
Selective visualization of intracellularly translocated proteins. Schematic illustration of the ferrocenylation of plasma membrane proteins and visualization of those proteins using Cy3-CB[7] and confocal laser scanning microscope images. **a** COS7 cells treated with **FcA-COOH**, EDC, and sulfo-NHS at 4 °C for 30 min, **b** the cells further incubated with **BAFc** at 4 °C for 20 min after **a**, and **c** the cells treated with **BAFc** at 4 °C for 20 min after additional incubation for 1 h at 37 °C of the cells in **a**. Scale bar = 10 μm

Since many dynamic cellular processes occur at the cell membrane, such as clustering and endocytosis, visualization of plasma membrane proteins is important. We have already demonstrated that plasma membrane proteins can be selectively ferrocenylated with **FcA-COOH** through EDC coupling at 4 °C at which intracellular uptake of **FcA-COOH** was significantly reduced^25^. To test whether proteins on plasma membranes are selectively visualized using the CB[7]-based ultrastable binding pair, live COS7 cells were treated with **FcA-COOH** as previously reported^25^, then the cells were treated with Cy3-CB[7] (100 nM). Confocal laser scanning microscopy revealed that the signals from Cy3-CB[7] were mostly observed at the periphery of the cells (Fig. 3a), which indicated the successful supramolecular latching of Cy3-CB[7] to the ferrocenylated proteins on the plasma membrane. Hardly any fluorescence signals (Supplementary Figure 6) were observed without **FcA-COOH** treatment, suggesting that the ultrastable host–guest interaction between Cy3-CB[7] and **FcA** moiety is crucial for the imaging of live cells, and that Cy3-CB[7] (100 nM) rarely adsorb non-specifically to the surface of the live cells.

### Selective visualization of proteins translocated into cytosol

Visualization of the translocation of plasma membrane proteins, such as epidermal growth factor receptor (EGFR) and G-protein coupled receptor (GPCR), in cells is necessary to understand their role in complex and dynamic processes such as endocytosis. By controlling the binding affinity of the host–guest complex, we selectively visualized membrane proteins that had been translocated into the cytosol. This was achieved by selectively unlatching Cy3-CB[7] from FcA-labeled proteins on the cell surface, while Cy3-CB[7]-latched proteins inside the cells were left untouched. The cells were treated with **BAFc** (100 μM), at 4 °C, which has approximately 3 orders higher binding affinity to CB[7] compared to that of **FcA** moiety^14–16^, which causes Cy3-CB[7] to unlatch from the **FcA**. The treatment almost completely abolished the fluorescence signal from Cy3-CB[7] (Fig. 3b) suggesting that Cy3-CB[7] was unlatched from the **FcA**-labeled proteins on the cell surface. After demonstrating the unlatching of Cy3-CB[7] from the cell surface proteins, we proceeded to selectively visualize translocated proteins. Another sample of cells labeled with Cy3-CB[7] was further incubated at 37 °C for 1 h to promote translocation of proteins, as a part of the normal metabolism of living cells. After treatment with **BAFc** at 4 °C, the fluorescence signal of Cy3-CB[7] was only visible inside the cells (Fig. 3c). These results indicated that we were observing the translocation of Cy3-CB[7] anchored plasma membrane proteins into the cells. In addition, the cells exhibited no significant morphological changes during the course of the experiment suggesting that the supramolecular latching system is not severely cytotoxic. Taken together, the conditional supramolecular unlatching of Cy3-CB[7] from the FcA labeled proteins on living cells, by guest exchange with a strong competitor (**BAFc**), allowed us to visualize, selectively, the proteins that underwent intracellular translocation.

### Visualization of intracellular proteins

**Fig. 4:**
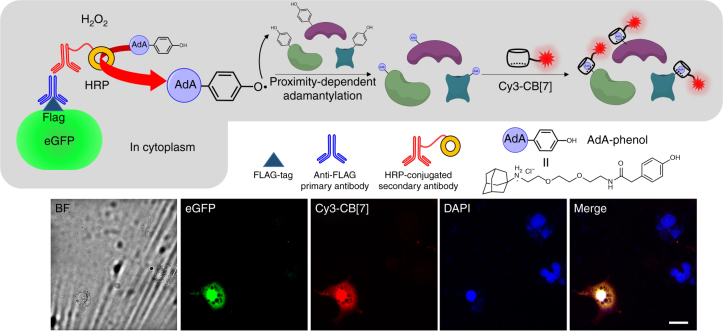
Visualization of intracellular proteins. Schematic illustration for adamantylation on cytoplasmic proteins using immunospecifically introduced HRP on eGFP and fluorescence microscopy images of the fixed COS7 cells which have FLAG-eGFP expressed in cytoplasm. Scale bar = 20 μm

Visualization of intracellular proteins is also important since most proteins are found within the cell. We used COS7 cells that were transfected (Lipofectamine^TM^ 2000) with plasmid DNA for the expression of FLAG^®^-tagged enhanced green fluorescent protein (FLAG-eGFP) in the cytoplasm. FLAG is a polypeptide protein tag (DYKDDDDK) which can be selectively recognized by a corresponding antibody (anti-FLAG primary antibody)^31^. After fixation of the cells with formaldehyde, the anti-FLAG primary antibody and a HRP-conjugated secondary antibody were sequentially treated to the cells for immunospecific introduction of HRP to the cytoplasmic FLAG-eGFP. When the fixed cells were treated with **AdA-phenol** (Supplementary Figure3) and H_2_O_2_, the HRP converted the phenol moiety of **AdA-phenol** to a phenyloxy radical, which then reacted with tyrosine residues on nearby proteins, thereby labeling the proteins with AdA (Fig. 4)^32^. After quenching the remaining radicals with sodium azide and sodium ascorbate, the cells were treated with fluorescent dyes, namely 4′,6-diamidine-2′-phenylindole dihydrochloride (DAPI) and Cy3-CB[7] for fluorescence staining of each cell and the AdA-labeled proteins in the cells, respectively.

Fluorescence images (Fig. 4) showed the appearance of a green fluorescence signal spread over the whole cell which indicated that FLAG-eGFP was well expressed in the cytoplasm of the transfected cells. However, some of the cells that were stained with DAPI, showing blue fluorescence, did not emit the green fluorescence signal. This indicated that not every cell is transfected. Pleasingly, when the cells were treated with Cy3-CB[7], only the FLAG-eGFP expressed cells showed good overlap of the fluorescence signals from eGFP with that from Cy3-CB[7]. The non-transfected cells rarely showed any signal from Cy3-CB[7] since FLAG-eGFP was not present. This result suggested that adamantylation only took place in the presence of the HRP-secondary antibody, which, in turn, was only present where the primary anti-FLAG and eGFP were. As a control experiment, we also used cells that were expressing eGFP without a FLAG-tag, we observed negligible fluorescence signals from Cy3-CB[7] (Supplementary Figure 7), which indicated that no Cy3-CB[7] was present due to the absence of AdA in the cells. This result supports that the specific adamantylation in Fig. 4 was facilitated by the FLAG-tag-based immunospecifically anchored HRP-secondary antibody. In addition, the absence of a significant signal caused by non-specific interactions of Cy3-CB[7] in the fixed cells is encouraging for its potential use as a cellular imaging tool. Taken together, intracellular proteins can be labeled with AdA using immunospecifically anchored enzymes and the adamantylated proteins can be efficiently visualized with Cy3-CB[7] using the highly selective and ultrastable host–guest interaction between CB[7] and AdA, without interference by endogenous biomolecules.

### Visualization of spatially resolved proteins

**Fig. 5:**
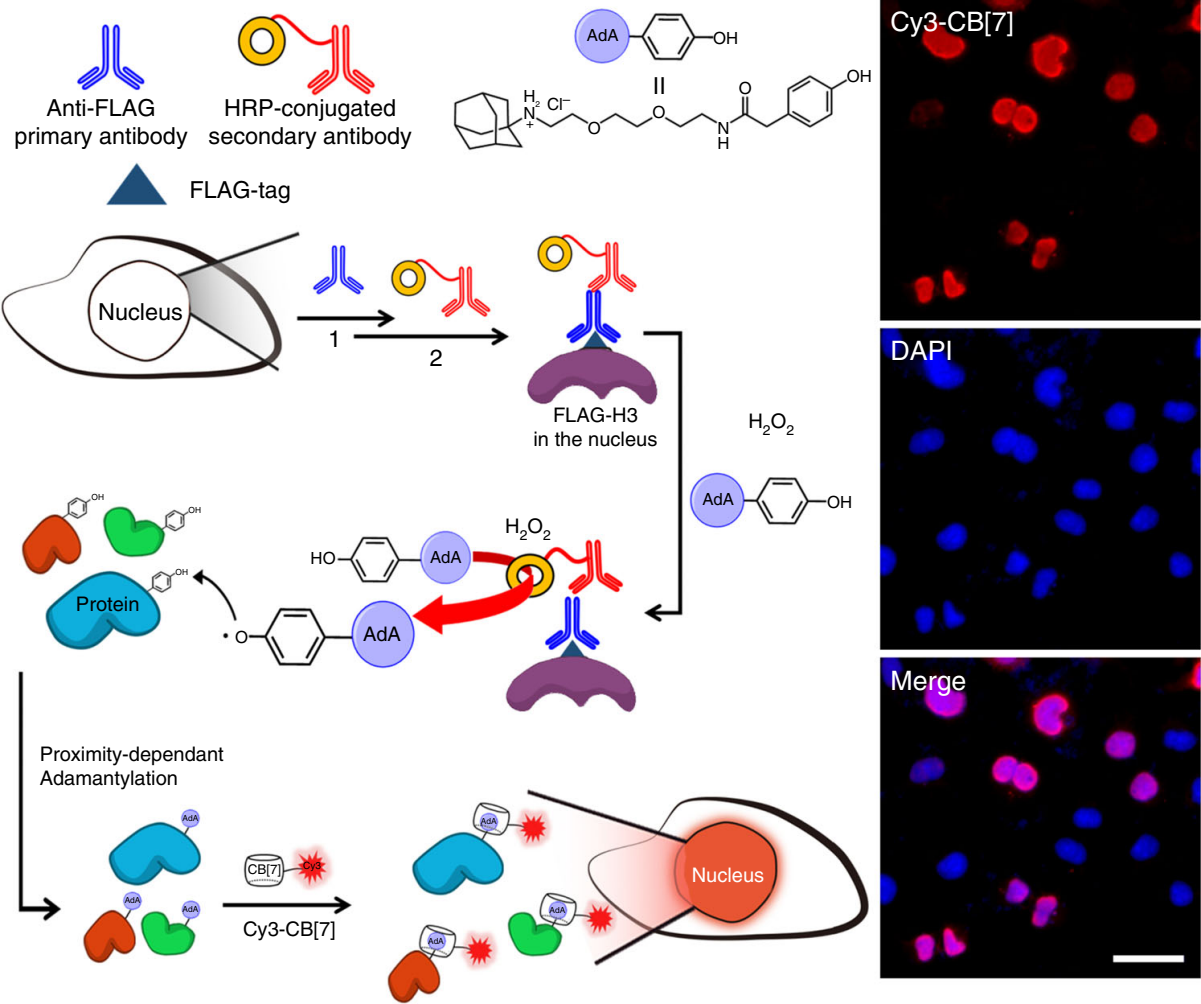
Visualization of nuclear proteins. Schematic illustration for proximity-dependent adamantylation of nuclear proteins using immunoselectively introduced HRP on histone H3 and fluorescence microscopy images of the fixed COS7 cells which have FLAG-histone H3 expressed in the nucleus. Scale bar = 20 μm

**Fig. 6:**
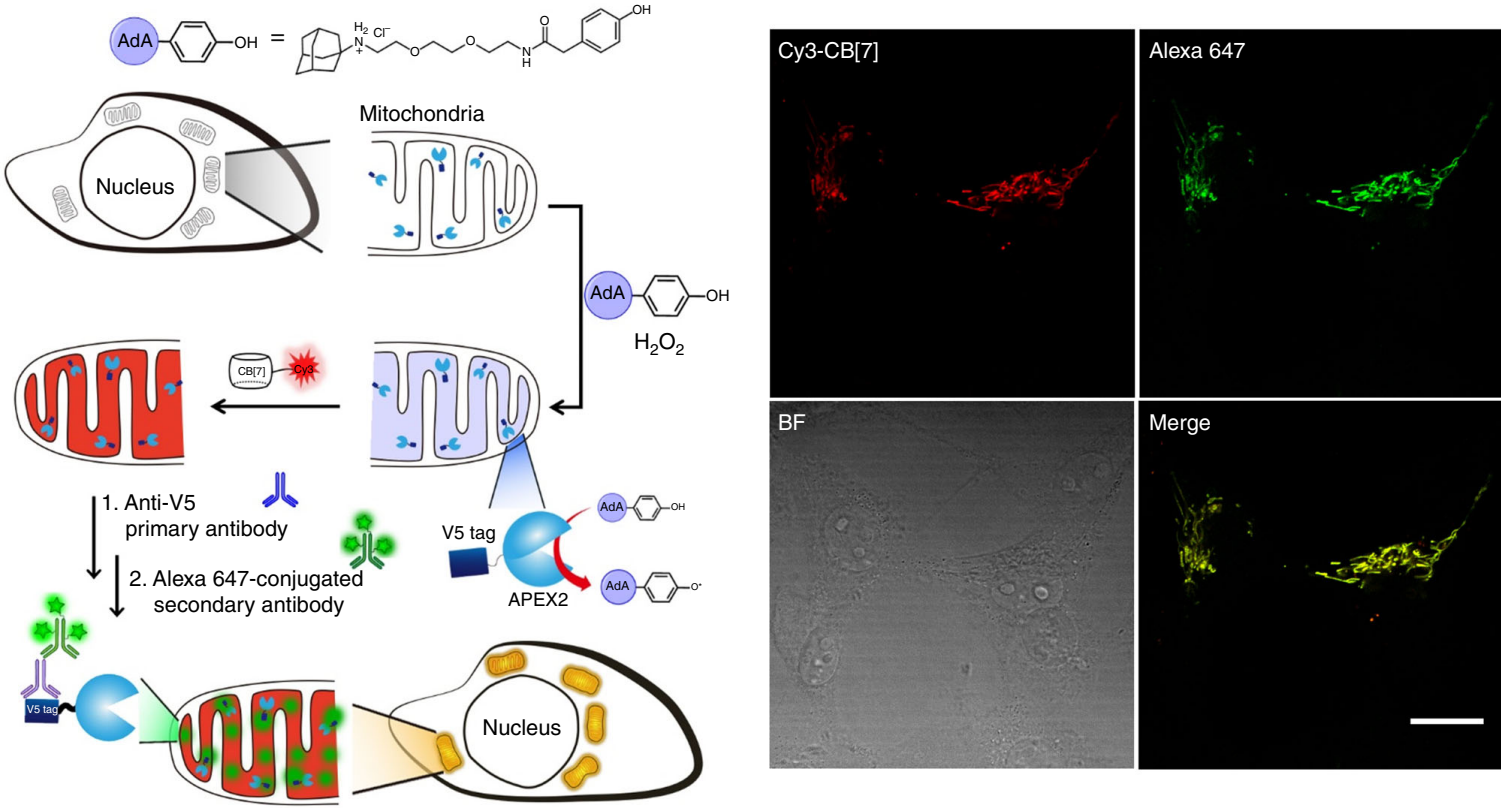
Visualization of a subcellular organelle (mitochondria). Schematic illustration for proximity-dependent adamantylation to mitochondria proteins using mito-V5-APEX2 and immunofluorescence labeling of mito-V5-APEX2, and confocal laser scanning microscopy images of living COS7 cells which have mito-V5-APEX2 expressed in mitochondria. Scale bar = 20 μm

Cells are composed of diverse specialized subunits, namely organelles, each of which is usually compartmentalized by a lipid membrane to regulate intracellular trafficking of proteins and perform metabolic functions^33^. To understand the role of these subunits in cells, visualization of spatially restricted proteins in such compartments is crucial. Thus, we examined whether the CB[7]-based ultrastable binding pair could ‘latch’ Cy3-CB[7] to AdA-labeled proteins in specific cellular organelles, thereby visualizing proteins in these compartments. To achieve this, we used two different proximity-dependent protein adamantylation approaches: (1) an immunospecifically introduced peroxidase (HRP) for fixed cells (Fig. 5); and (2) an engineered APEX2 for living cells (Fig. 6)^34^. For proximity-dependent adamantylation of proteins in fixed cells, we used a similar strategy as in the previous section. In this case, we immunospecifically introduced HRP to the nucleus of COS7 cells by expressing FLAG^®^-tagged histone H3 protein (FLAG-H3). Before performing HRP-induced adamantylation, we first confirmed the successful expression of FLAG-H3 in the nucleus of the cells by observing well-overlapped fluorescence signal of DAPI (a staining dye for the cell nucleus) with that of immunospecifically labeled Alexa 555-conjugated secondary antibody, which specifically recognizes anti-FLAG primary antibody (Supplementary Figure 8). The COS7 cells were fixed and sequentially treated with anti-FLAG primary antibody, HRP-conjugated secondary antibody, and **AdA-phenol** with H_2_O_2_ by following the same procedure as we performed for the visualization of cytoplasmic proteins with FLAG-eGFP. After the cells were treated with DAPI and Cy3-CB[7], fluorescence microscopy was used to examine the location of the AdA-labeled proteins. The signal from Cy3-CB[7] was well-overlapped with that of DAPI (Fig. 5), suggesting that the proteins were labeled exclusively in the nucleus and Cy3-CB[7] latches onto those labeled proteins selectively. In summary, the combination of proximity-based labeling and an ultrastable host–guest interaction between Cy3-CB[7] and AdA allows visualization of the proteins in compartments deep inside fixed cells.

Organelle-specific protein labeling in living cells can be achieved by using an engineered APEX2. Although APEX2 and HRP seem to work similarly in terms of reaction chemistry, HPR does not have sufficient reactivity in living cells, but APEX2 does^34^. We investigated whether APEX2 could be used to label proximal proteins with AdA and then see if Cy3-CB[7] could visualize those proteins. We ectopically expressed the mito-V5-APEX2 fusion protein which was designed to translocate into the mitochondrial matrix in COS7 cells. The V5 epitope (a polypeptide protein tag-GKPIPNPLLGLDST) allowed visualization of the protein using anti-V5 antibody for immunospecific fluorescence labeling^35^. Protein labeling inside the organelle of the living cells was initiated by treatment with **AdA-phenol** (for 30 min) and H_2_O_2_ (1.0 mM for 2 min, which is endurable for living cells)^34^. Immediately after quenching the remaining radicals with sodium azide and sodium ascorbate, the cells were fixed and treated with Cy3-CB[7] to visualize the AdA-labeled proteins in the cells. In parallel, we performed immunofluorescence labeling with anti-V5 primary antibody and Alexa647-conjugated secondary antibody to confirm the subcellular localization of mito-V5-APEX2 enzyme in the cells. Using confocal laser scanning microscopy, we observed the fluorescence signal corresponding to the immunospecific labeling of the V5-epitope (Fig. 6). The signal appeared as a punctuated structure which is a characteristic structural feature of mitochondria, suggesting that mito-V5-APEX2 is well expressed and located in the target organelle. We also observed the signal corresponding to Cy3-CB[7] and found that the signal was well-overlapped with that of immunofluorescence signal of V5 (Fig. 6). These results showed that spatially restricted adamantylation of the proteins in a specific subcellular organelle in living cells can be performed using mito-V5-APEX enzyme and the adamantylated proteins can be specifically visualized with Cy3-CB[7] via the ultrastable host–guest interactions between CB[7] and AdA. In addition, we found that the cells required at least 25 μM of **AdA-phenol** treatment for efficient enzyme-assisted proximity-dependent protein adamantylation to visualize the target proteins with Cy3-CB[7]; however, 250 μM of **AdA-phenol** seemed to be optimum for the conditions that we tested in this study.

### Visualization of target-specific proteins

**Fig. 7:**
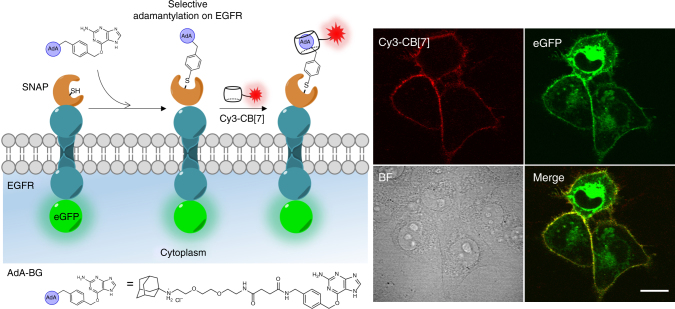
Visualization of a target specific protein. Schematic illustration of adamantylation on r-EGFR using SNAP-tag method. Confocal laser scanning microscopy images of COS7 cells which have SNAP and eGFP-tagged recombinant EGFR expressed in the plasma membrane. Scale bar = 20 μm

To understand the roles of specific proteins involved in cellular functions, visualization of specific proteins in living cells is of a great interest in chemical biology^36–39^. For this purpose, we investigated whether the supramolecular latching system could be exploited for the imaging of specific target proteins in living cells (Fig. 7). SNAP-tag is a mutant of the DNA-repair protein, O^6^-alkylguanine-DNA alkyltransferase, and can be ectopically expressed as a fusion protein with the target protein. SNAP-tag reacts spontaneously with benzylguanine (BG) derivatives to make a covalent conjugation of BG derivative to the SNAP-tag-fusion protein. Into living cells, we transfected a DNA construct encoding a plasma membrane receptor that contains an *N*-terminal SNAP and *C*-terminal eGFP-tagged EGFR (SNAP and eGFP recombinant EGFR, r-EGFR). After transfection, the cells were incubated with a SNAP-reactive AdA molecule, **AdA-BG** (Supplementary Figure 4), and then treated with Cy3-CB[7]. Confocal laser scanning microscopy revealed that the fluorescence signals from Cy3-CB[7] mainly arose from the surface of the cells, which is well-overlapped with that from eGFP. (Fig. 7) This result suggested that the cell surface-exposed SNAP-tag on r-EGFR was specifically adamantylated with **AdA-BG** and Cy3-CB[7] was successfully latched to the adamantylated protein through the ultrastable host–guest chemistry.

### Visualization of proteins on a living animal (*C. elegans*)

**Fig. 8:**
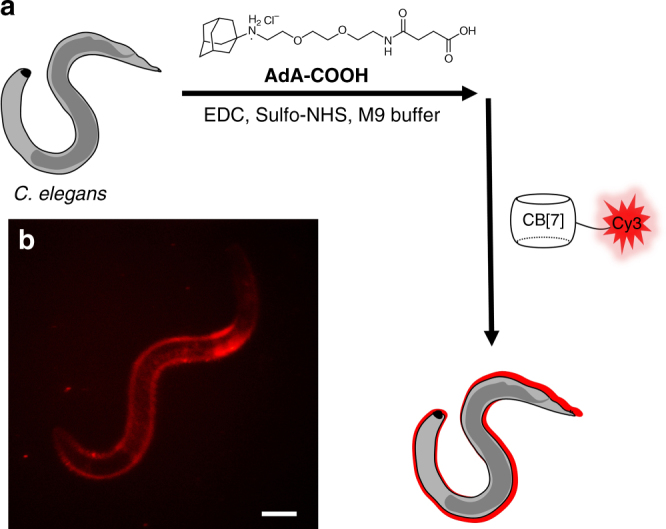
Visualization of a simple animal model (*C. elegans*). **a** Schematic illustration of adamantylation on *C. elegans* and **b** fluorescence microscopy images of *C. elegans* after treating with Cy3-CB[7]. Scale bar = 50 μm

Not limited to cell-level imaging, we investigated whether the supramolecular latching system can be extended to animal-level imaging. As a simple animal model, we choose *C. elegans*, which is a nematode (roundworm) about 1 mm in length when it becomes an adult. Owing to easy cultivation, well-known anatomical information, and transparency, *C. elegans* have been widely used for fluorescence bioimaging studies of multicellular organisms. After treating *C. elegans *with **AdA-COOH** (see Supplementary Figure 4), activated with *N*-hydroxysulfosuccinimide sodium salt (sulfo-NHS) and EDC, we then treated with Cy3-CB[7] and imaged it under a fluorescence microscope. The fluorescence signal from Cy3-CB[7] appeared mostly on the surface of *C. elegans* (Fig. 8), whereas almost no fluorescence was observed from *C. elegans* that was not treated with **AdA-COOH** (Supplementary Figure 9). These results indicated that **AdA-COOH** seemed to be conjugated to the proteins mostly on the surface of *C*. *elegans* and Cy3-CB[7] efficiently latched onto the proteins to be visualized. Taken together, CB[7]-based ultrastable binding pairs are potentially useful as a supramolecular latching system even for animal-level imaging.

## Discussion

We have demonstrated that small, non-protein-based synthetic binding pairs can act as a switchable supramolecular latching system for the fluorescence imaging of proteins located in and on fixed or living cells. These pairs are not only a replacement of SA–BT, they are superior on account of their bioorthogonality in binding, controllability of their binding, chemical stability, and small size. Proteins in (or on) the cells were adamantylated (or ferrocenylated) using various labeling approaches such as direct chemical labeling through EDC coupling, enzyme-assisted labeling with immunospecifically introduced peroxidase (HRP), genetically expressed peroxidase (APEX2), and a self-labeling protein (SNAP-tag). Cy3-CB[7] can be latched to the adamantylated (or ferrocenylated) proteins by taking advantage of the ultrastable host–guest interaction between Cy3-CB[7] and AdA (or FcA). Successful visualization of the proteins with cells and *C. elegans* suggests that the supramolecular latching system has great potential for various living organism bioimaging, from cells to animals. We observed exceptional selectivity of CB[7] to AdA (or FcA) with no interference from endogenous biomolecules enabling us to have clear fluorescence images for accurate and precise analysis of protein locations using fluorescence microscopy. This is in contrast to SA–BT, which can be subject to interference from endogenous biotin and biotinylated proteins. Conditional unlatching of Cy3-CB[7] from FcA (or AdA)-labeled proteins on living cells membrane by guest exchange allowed us to selectively visualize intracellularly translocated proteins. Moreover, no significant non-specific adsorption of Cy3-CB[7] onto the cells or *C. elegans* was observed. The concentration range of Cy3-CB[7] (between 25 nM and 100 nM) used in the cell and *C. elegans* experiments seems to be the optimal range for the reagent. When the concentration of Cy3-CB[7] is less than 25 nM, visualization of proteins in cells and animals is difficult to achieve. On the other hand, when more than 100 nM is used, the background signal can be high, especially in the case of fixed cells. These results can serve as starting points in the optimization of treatment conditions for other protein imaging assays with cells and animals. Taken together, the CB[7]-based system reported here demonstrates the great potential of the ultrastable synthetic binding pairs as a new tool for cell and animal-level bioimaging. The small size of these pairs (relative to proteins) and their ability to selectively label proteins of interest with FcA (or AdA), using chemical or genetic approaches, may allow for super-resolution imaging of proteins and their dynamic behaviors such as vesicle trafficking; work along this line is currently underway.

## Methods

### Transfection of genes to the cells

COS7 cells were cultured in DMEM supplemented with 10% (v/v) fetal bovine serum at 37 °C in a humidified CO_2_-controlled (5%) incubator. For transfection and transient expression of proteins, the cells were transfected with plasmids encoding a recombinant gene using Lipofectamine^TM^ 2000 (Invitrogen, Carlsbad, CA). According to the manufacturer’s instructions, transfections were performed and the cells were then cultured for an additional 24 h to achieve ectopic expression of interest proteins.

### Visualization of AdA-labeled proteins with Cy3-CB[7]

The COS7 cells cultured on a circular cover glass (16 mm in diameter) were cultured in DMEM (1.0 ml) supplemented with 10% fetal bovine serum at 37 °C under 5% CO_2_. The cells were fixed with 4% formaldehyde in phosphate-buffered saline (PBS) (1.0 ml) at room temperature for 15 min and washed with PBS (5×, 1.0 ml) and permeabilized with a solution of 0.5% Triton X-100 in PBS (1.0 ml) for 5 min at room temperature. The cells were washed again (3×) with PBS (1.0 ml) at room temperature and treated with a solution of 3% bovine serum albumin (BSA) in Tris-buffered saline with 0.1% of Tween 20 (TBST) (1.0 ml). The cells were stained with anti-golgin97 primary antibody (Invitrogen, 1:1000) in the blocking buffer for 1 h at room temperature and washed with PBS (5×, 1.0 ml).

Golgin97 imaging using AdA-secondary antibody: The AdA-conjugated secondary goat anti-mouse antibody (1:300), Cy3-CB[7] (25 nM), and secondary anti-mouse-AF647 antibody (Invitrogen 1:1000) were mixed in PBS (50 µl). The mixture was purified by centrifugal filtration (MWCO, 30 kDa) with PBS to obtain the antibody mixture solution. After mixing with a blocking buffer (450 µl), the solution was introduced to the cells to stain the golgin97 for confocal laser scanning microscopy.

Golgi97 imaging using BT-secondary antibody: The BT-conjugated secondary goat anti-mouse antibody (Invitrogen, 1:8000) and secondary anti-mouse-AF555 antibody (Invitrogen, 1:1000) were mixed in blocking buffer. The cells were stained with the mixture for 1 h at room temperature. After washing (5×, 1.0 ml) with a solution of TBST, the cells were stained with AF647-SA (Invitrogen, 1:1000) for 1 h at room temperature for confocal laser scanning microscopy. The cells on the cover glass were examined by a confocal laser scanning microscope system (LSM 700, Zeiss) with 63×/1.40 NA Oil objective.

The confocal laser scanning microscopy images (Fig. 2a–h) were used for calculation of colocalization efficiency (%) between AF647-SA and AF555-secondary antibody, and Cy3-CB[7] and AF647-secondary antibody. The numbers (#) of the fluorescently stained pixels and their overlapping were obtained from a imaging software (ZEN, Zeiss). The fluorescence signals of AF555- and AF647-secondary antibodies were almost included in those of AF647-SA and Cy3-CB[7], respectively (Supplementary Figure 5). The values for the colocalization efficiency (see Supplementary Table 2) were calculated by (*N*_Ov1_/*N*_AF647-SA_) × 100 and (*N*_Ov2/_*N*_Cy3-CB[7]_) × 100 with five different cells for target protein imaging with AF647-SA and Cy3-CB[7], respectively. The closer the values were to 100%, the more precise and accurate imaging of the target protein (Golgi 97).

# of pixels overlapping between AF647-SA and AF555-secondary antibody = *N*_Ov1_

# of pixels stained with AF647-SA for BT-secondary antibody = *N*_AF647-SA_

# of pixels overlapping between Cy3-CB[7] and AF647-secondary antibody = *N*_Ov2_

# of pixels stained with Cy3-CB[7] for AdA-secondary antibody = *N*_Cy3-CB[7]_

### Visualization of FcA-labeled proteins

COS7 cells were cultured on a circular cover glass (16 mm in diameter) in a 24-well plate to 70–80% confluency and briefly washed with ice-cold PBS three times before the treatment of labeling reagents. The cells in the culture medium (1.0 ml) were treated with (or without) a solution of **FcA-COOH** (0.4 mg)^25^, sulfo-NHS (0.3 mg), and EDC (0.3 mg) in PBS (10 μl), and then incubated for 30 min at 4 °C, followed by washing with ice-cold PBS three times. The cells were additionally incubated in a solution of Cy3-CB[7] (100 nM) in PBS (1.0 ml) for 10 min at 4 °C and washed with PBS (1.0 ml) three times. The cells applied with a drop of VECTASHIELD^®^ (an antifade mounting medium with DAPI) on the cover glass were examined by a confocal laser scanning microscope system (LSM 700, Zeiss) with 63×/1.40 NA Oil objective.

### Supramolecular latch-off of Cy3-CB[7] from the FcA protein

The cells with Cy3-CB[7] latched to the plasma membrane proteins, prepared as above, were incubated with **BAFc** (2 μl, 10 mM) for 20 min in the culture medium (200 μl) at 4 °C. After washing the cells with PBS (1.0 ml) three times, the cells applied with a drop of VECTASHIELD^®^ on the cover glass were examined by confocal laser scanning microscope system (LSM 700, Zeiss) with 63×/1.40NA Oil objective.

### Visualization of intracellularly translocated proteins

The COS7 cells with Cy3-CB[7] on the surface, prepared as above, were further incubated at 37 °C for 2 h and washed with PBS (1.0 ml) three times. The washed cells were treated with BAFc and further incubated for 20 min at 4 °C. The cells were washed with PBS (1.0 ml) three times and applied with a drop of VECTASHIELD^®^ on the cover glass were examined by confocal laser scanning microscope system (LSM 700, Zeiss) with 63×/1.40NA Oil objective.

### Immunofluorescence labeling

The COS7 cells cultured on a circular cover glass (16 mm in diameter) treated with samples were fixed in a solution of paraformaldehyde (4%) in PBS (1.0 ml) at room temperature for 15 min. Then, the cells were washed three times with PBS and permeabilized with a solution of Triton X-100 (0.5 %) in PBS (1.0 ml) for 15 min. After incubation with a solution of BSA (3%) in PBS (1.0 ml) as a blocking buffer for 15 min, the cells were stained with anti-FLAG (Sigma-Aldrich, 1:1000 dilution) or anti-V5 mouse antibodies (Invitrogen; 1:1000 dilution) in the blocking buffer (1.0 ml) overnight, and then washed five times with a solution of Tween-20 (0.1%) in PBS (PBST) (1.0 ml). After this, the cells were either processed for immunofluorescence or for immunospecifically proximate protein labeling. For immunofluorescence imaging, the cells were incubated with a solution of Alexa488-conjugated anti-mouse IgG secondary antibody (Invitrogen; 1:1000 dilution) in the blocking buffer (1.0 ml) for 1 h at room temperature. After five washings with PBST, the cells were examined under an epi-fluorescence microscope as previously described for the imaging of the cell surface proteins.

For immunospecifically proximity-dependent protein labeling, the cells were incubated with a solution of HRP-conjugated anti-mouse IgG secondary antibody (Cell Signaling Technology; 1:1000 dilution) in the blocking buffer (1.0 ml) for 3 h at room temperature. After five washings with PBST (1.0 ml), the cells were treated with **AdA-phenol** (250 μM), H_2_O_2_ (1 mM) in PBS (1.0 ml) for 2 min. Thereafter, the reaction was quenched with a solution of sodium azide (10 mM) and sodium ascorbate (10 mM) in PBS (2 ml). The cells were incubated in the blocking buffer (1.0 ml) for 15 min and then treated with Cy3-CB[7] (100 nM) prepared in the blocking buffer (1.0 ml) for 30 min at room temperature. After five washings with PBST (1.0 ml), the cells were examined under an epi-fluorescence microscope as previously described for the imaging of cell surface proteins.

### APEX2-dependent protein labeling and imaging

After the transfection of mito-V5-APEX2 to COS7 cells on a circular cover glass (16 mm in diameter), the cells were treated with **AdA-phenol** (250 μM) in a cell culture medium (1.0 ml) and incubated for 30 min at 37 °C, then treated with H_2_O_2_ (1.0 ml, 1 mM) for 2 min to initiate adamantylation. The reaction was quenched with a solution of sodium azide (5 mM) and sodium ascorbate (5 mM) in PBS (2.0 ml). Thereafter, the cells were fixed in a solution of paraformaldehyde (4%) in PBS (1.0 ml) at room temperature for 15 min. The fixed cells were washed three times with PBS (1.0 ml) and permeabilized with a solution of Triton X-100 (0.5 %) in PBS (1.0 ml) for 15 min. After incubation with the blocking buffer (2.0 ml) for 15 min, the cells were incubated with a solution of anti-V5 primary antibody (Invitrogen; 1:1000 dilution) in the blocking buffer solution (1.0 ml) for 8 h and then washed five times with PBS (1.0 ml). The washed cells were incubated with a solution of Cy3-CB[7] (50 nM) and Alexa647-conjugated anti-mouse IgG secondary antibody (Invitrogen; 1:1000 dilution) in the blocking buffer (1.0 ml) for 1 h at room temperature. After further five washings with PBS (1.0 ml), the cells on the cover glass were examined under a confocal laser scanning microscope (FV1000, Olympus) with 60×/1.42 Oil objective.

### SNAP-based specific protein labeling and imaging

After the transfection of *N*-terminal SNAP and *C*-terminal eGFP-tagged EGFR, the cells on a cover glass (16 mm in diameter) were treated with a solution of **AdA-BG** (10 μM) in a serum-free cell culture medium (1.0 ml) and incubated for 30 min at 37 °C. Then, the cells were washed with PBS three times and treated with a solution of Cy3-CB[7] (200 nM) in a blocking buffer (1.0 ml) for another 30 min at room temperature. After five times washings with PBS, the cells on a cover glass were examined under confocal laser scanning microscope (FV1000, Olympus) with 60×/1.42NA Oil objective.

### Protein labeling and imaging on *C. elegans*

*C. elegans* (wild-type N_2_) was provided by Caenorhabditis Genetics Center (CGC), University of Minnesota, and maintained as required. Briefly, *C. elegans* was cultured on solid Nematode Growth Medium (NGM) plates and fed with a lawn of OP50 (a uracil auxotrophic *Escherichia coli* strain which grows slowly on NGM plates) at room temperature. For all experiments, the eggs of *C. elegans* were isolated in advance and collected in M9 buffer, and then L1 stage larvae are hatched after incubation at room temperature for 24 h. After ca. 100 newly hatched L1 larvae were briefly washed with M9 buffer (1.0 ml) three times, the larvae were treated with a solution (50 μl) of **AdA-COOH** (1.0 mg), sulfo-NHS (0.7 mg), and EDC (0.7 mg) in M9 buffer (1.0 ml) and incubated for 4 h at 4 °C. After washing with an ice-cold M9 buffer, the larvae were incubated in a solution of Cy3-CB[7] (100 nM) in M9 buffer (50 μl) for 10 min at room temperature. After another washing step with ice-cold M9 buffer, the *C. elegans* were placed on an agar-coated slide glass and examined under an epi-fluorescence microscope (Eclipse Ti-E, Nikon) with 20×/0.75 NA objective.

### Data availability

The authors declare that the main data supporting the findings of this study are available within the article and its Supplementary Information files. Extra data are available from the corresponding author upon request.

## Electronic supplementary material

Supplementary Information

## References

[1] Swamy MJ (1995). Thermodynamic analysis of biotin binding to avidin. A high sensitivity titration calorimetric study. Biochem. Mol. Biol. Int..

[2] Howarth M, Ting AY (2008). Imaging proteins in live mammalian cells with biotin ligase and monovalent streptavidin. Nat. Protoc..

[3] Reimann O, Smet-Nocca C, Hackenberger CPR (2015). Traceless purification and desulfurization of tau protein ligation products. Angew. Chem. Int. Ed..

[4] Sreekanth KV (2016). Extreme sensitivity biosensing platform based on hyperbolic metamaterials. Nat. Mater..

[5] Berntzen G (2006). Characterization of an Fc gamma RI-binding peptide selected by phage display. Protein Eng. Des. Sel..

[6] Jungmann R (2014). Multiplexed 3D cellular super-resolution imaging with DNA-PAINT and Exchange-PAINT. Nat. Methods.

[7] Bussolati G, Gugliotta P, Volante M, Pace M, Papotti M (1997). Retrieved endogenous biotin: a novel marker and a potential pitfall in diagnostic immunohistochemistry. Histopathology.

[8] Rusckowski M, Fogarasi M, Fritz B, Hnatowich DJ (1997). Effect of endogenous biotin on the applications of streptavidin and biotin in mice. Nucl. Med. Biol..

[9] Banks RE, Craven RA, Harnden PA, Selby PJ (2003). Use of a sensitive EnVision (TM)+-based detection system for Western blotting: avoidance of streptavidin binding to endogenous biotin and biotin-containing proteins in kidney and other tissues. Proteomics.

[10] Tytgat HLP (2015). Endogenous biotin-binding proteins: an overlooked factor causing false positives in streptavidin-based protein detection. Microb. Biotechnol..

[11] Horling L, Neuhuber WL, Raab M (2012). Pitfalls using tyramide signal amplification (TSA) in the mouse gastrointestinal tract: endogenous streptavidin-binding sites lead to false positive staining. J. Neurosci. Methods.

[12] Lee, J. W., Samal, S., Selvapalam, N., Kim, H. -J. & Kim, K. Cucurbituril homologues and derivatives: new opportunities in supramolecular chemistry. *Acc. Chem. Res.* **36**, 621–630 (2003). 10.1021/ar020254k 12924959

[13] Barrow SJ, Kasera S, Rowland MJ, del Barrio J, Scherman OA (2015). Cucurbituril-based molecular recognition. Chem. Rev..

[14] Jeon WS (2005). Complexation of ferrocene derivatives by the cucurbit[7]uril host: a comparative study of the cucurbituril and cyclodextrin host families. J. Am. Chem. Soc..

[15] Liu SM (2005). The cucurbit[n]uril family: prime components for self-sorting systems. J. Am. Chem. Soc..

[16] Rekharsky MV (2007). A synthetic host-guest system achieves avidin-biotin affinity by overcoming enthalpy-entropy compensation. Proc. Natl Acad. Sci. USA.

[17] Moghaddam S (2011). New ultrahigh affinity host-guest complexes of cucurbit[7]uril with bicyclo[2.2.2]octane and adamantane guests: thermodynamic analysis and evaluation of M2 affinity calculations. J. Am. Chem. Soc..

[18] Cao LP (2014). Cucurbit[7]uril.guest pair with an attomolar dissociation constant. Angew. Chem. Int. Ed..

[19] Isaacs L (2014). Stimuli responsive systems constructed using cucurbit[n]uril-type molecular containers. Acc. Chem. Res..

[20] Assaf KI, Nau WM (2015). Cucurbiturils: from synthesis to high-affinity binding and catalysis. Chem. Soc. Rev..

[21] Shetty D, Khedkar JK, Park KM, Kim K (2015). Can we beat the biotin-avidin pair?: cucurbit[7]uril-based ultrahigh affinity host-guest complexes and their applications. Chem. Soc. Rev..

[22] Park KM, Murray J, Kim K (2017). Ultrastable artificial binding pairs as a supramolecular latching system: a next generation chemical tool for proteomics. Acc. Chem. Res..

[23] Lee DW (2015). A simple modular aptasensor platform utilizing cucurbit[7]uril and a ferrocene derivative as an ultrastable supramolecular linker. Chem. Commun..

[24] Ahn Y, Jang Y, Selvapalam N, Yun G, Kim K (2013). Supramolecular velcro for reversible underwater adhesion. Angew. Chem. Int. Ed..

[25] Lee DW (2011). Supramolecular fishing for plasma membrane proteins using an ultrastable synthetic host-guest binding pair. Nat. Chem..

[26] Tonga GY (2015). Supramolecular regulation of bioorthogonal catalysis in cells using nanoparticle-embedded transition metal catalysts. Nat. Chem..

[27] Kim C, Agasti SS, Zhu ZJ, Isaacs L, Rotello VM (2010). Recognition-mediated activation of therapeutic gold nanoparticles inside living cells. Nat. Chem..

[28] Gong B (2015). High affinity host-guest FRET pair for single-vesicle content-mixing assay: observation of flickering fusion events. J. Am. Chem. Soc..

[29] Bockus AT (2016). Cucurbit[7]uril-tetramethylrhodamine conjugate for direct sensing and cellular imaging. J. Am. Chem. Soc..

[30] Li M (2018). Autophagy caught in the act: a supramolecular FRET pair based on an ultrastable synthetic host-guest complex visualizes autophagosome-lysosome fusion. Angew. Chem. Int. Ed..

[31] Einhauer A, Jungbauer A (2001). The FLAG peptide, a versatile fusion tag for the purification of recombinant proteins. J. Biochem. Biophys. Methods.

[32] Minamihata K, Goto M, Kamiya N (2011). Site-specific protein cross-linking by peroxidase-catalyzed activation of a tyrosine-containing peptide tag. Bioconjug. Chem..

[33] Lowe M, Barr FA (2007). Inheritance and biogenesis of organelles in the secretory pathway. Nat. Rev. Mol. Cell Biol..

[34] Rhee HW (2013). Proteomic mapping of mitochondria in living cells via spatially restricted enzymatic tagging. Science.

[35] Casper JM (2005). The c-myc DNA-unwinding element-binding protein modulates the assembly of DNA replication complexes in vitro. J. Biol. Chem..

[36] Xue L, Karpenko IA, Hiblot J, Johnsson K (2015). Imaging and manipulating proteins in live cells through covalent labeling. Nat. Chem. Biol..

[37] Pratt CP, He JJ, Wang Y, Barth AL, Bruchez MP (2015). Fluorogenic green-inside red-outside (GIRO) labeling approach reveals adenylyl cyclase-dependent control of BK alpha surface expression. Bioconjug. Chem..

[38] Li CG (2017). Dynamic multicolor protein labeling in living cells. Chem. Sci..

[39] Fisher GW (2014). Self-checking cell-based assays for GPCR desensitization and resensitization. J. Biomol. Screen..

