# Supplementary material for: Supramolecular latching system based on ultrastable synthetic binding pairs as versatile tools for protein imaging
Source: Nat Commun. 2018 Apr 27;9:1712. doi: 10.1038/s41467-018-04161-4 (PMC5923385; doi:10.1038/s41467-018-04161-4)
Supplement: Supplementary file 1 — Supplementary Information [file 41467_2018_4161_MOESM1_ESM.pdf]

Supplementary Information

Supramolecular Latching System Based on Ultrastable Synthetic Binding Pairs as Versatile Tools for Protein Imaging

Kyung Lock Kim,[+] Gihyun Sung,[+] Jaehwan Sim, Meng Li, James Murray, Ara Lee, Annadka Shrinidhi, Kyeng Min Park,[*] Kimoon Kim[*]

Center for Self-assembly and Complexity (CSC), Institute for Basic Science (IBS), Pohang 37673, Republic of Korea, Division of Advanced Materials Science, Pohang University of Science and Technology (POSTECH), Pohang 37673, Republic of Korea, School of Interdisciplinary Bioscience and Bioengineering, Pohang University of Science and Technology (POSTECH), Pohang 37673, Republic of Korea, University of Science and Technology (UST), Department of Nanomaterials and Engineering, Daejeon 34113, Republic of Korea, Department of Chemistry, Pohang University of Science and Technology (POSTECH), Pohang 37673, Republic of Korea

[+]Kyung Lock Kim and Gihyun Sung contributed equally to this work

Correspondence should be addressed to K.M.P. (kmpark@ibs.re.kr) and K.K. (kkim@postech.ac.kr).

Supplementary Methods

General Procedures

All the reagents and solvents employed were commercially available and used as supplied without further purification. The nuclear magnetic resonance (NMR) spectra were acquired at 298 K on a Bruker Advance 850 MHz. High-resolution mass (electrospray ionization, ESI) data were obtained with a SYNAPT G2 (Waters, U.K.) mass spectrometer at the Korea Basic Science Institute (Ochang, Korea). Mass (MS) analysis was performed using a LTQ-XL mass spectrometer (Thermo Fisher Scientific, Inc.) equipped with an electrospray ionization (ESI) source. For automatic measurement and data analysis, Xcalibur software was used (Thermo Fisher Scientific, Inc.). Fluorescence images were collected with an epi-fluorescent microscope system (Eclipse Ti-E, Nikon) and confocal laser scanning microscopes (FV 1000, Olympus, LSM 700, Zeiss), *C. elegans* (wild-type N_2) was provided by Caenorhabditis Genetics Center (CGC), University of Minnesota, and maintained as required. Cy3-CB[7] and **FcA-COOH** are synthesized by following the previous reports.[1, 2] COS7 cell line and HeLa cell line was generously provided from Prof. Sung Ho Ryu (POSTECH)

Synthesis of N_3-COOH (Supplementary Figure 1)

1-Amino-8-azido-3, 6-dioxaoctane (50 mg, 0.29 mmol from FutureChem, Seoul, South Korea) was added to a solution of succinic anhydride (40 mg, 0.4 mmol) and trimethylamine (10 μl) in methylene chloride (MC, 50 ml) and stirred at room temperature for 8 hr. The reaction mixture was evaporated under the reduced pressure and purified by silica column chromatography (eluent, MC:MeOH = 10:1) to give N_3-COOH (40 mg, 50%). ^{1}H-NMR (850 MHz, D_2O): δ 3.76 – 3.72 (m, 6H), 3.66 (t, 2H), 3.53 (t, 2H), 3.43 (t, 2H), 2.66 (t, 2H), 2.57 (t, 2H). MS-ESI (m/z): $[M + H]^+$ calcd for $C_{10}H_{19}N_4O_5$, 275.13; found, 275.17.

Synthesis of AdA-DBCO (Supplementary Figure 2)

1[2] (50 mg, 0.18 mmol) was added to a solution of DBCO-C_6-NHS (60 mg, 0.14 mmol from Click Chemistry Tools, Scottsdale, AZ) in MC (10 ml) and stirred at room temperature for 8 hr. The reaction mixture was evaporated under the reduced pressure and purified by silica column chromatography (eluent, MC:MeOH:NEt_3 = 10:1:0.1)to give AdA-DBCO (40 mg, 49 %). ^{1}H-NMR (850 MHz, D_2O): δ 7.61 (d, 1H), 7.45 – 7.41 (m, 4H), 7.33-7.27 (m, 2H), 7.21 (d, 1H), 5.10 (d, 1H), 3.66 (d, 1H), 3.59 - 3.55 (m, 6H), 3.45 (t, 2H), 3.27 (m, 2H), 3.25 – 3.23 (m, 2H), 2.84 (t, 2H), 2.18 - 2.14 (m, 1H), 2.06 (m, 3H), 1.94 – 1.89 (m, 3H), 1.72 (s, 6H), 1.71 - 1.69 (m, 3H), 1.63- 1.62 (m, 3H), 1.35 – 1.32 (m, 1H), 1.29 – 1.22 (m, 3H). MS-ESI (m/z): $[M + H]^+$ calcd for $C_{37}H_{48}N_3O_4$, 598.36; found, 598.92.

Adamantanylation of Anti-Mouse secondary antibody (IgG)

N, N′-Diisopropylcarbodiimide (DIC, 0.1 mg, 0.9 μmol) and *N*-hydroxysulfosuccinimide sodium salt (Sulfo-NHS, 0.2 mg, 0.8 μmol) were added to a solution of N_3-COOH (0.2 mg, 0.6 μmol) in DMF (17 μl) and the reaction mixture was stirred at room temperature for 2 h. The crude product was directly added to a solution of the secondary goat anti-mouse antibody (IgG) (1 mg, 6.7 nmol) in PBS (180 μl) and stirred at 4 ºC for 15 minutes. The antibody solution was purified by centrifugal filtration (MWCO, 30 kDa) with PBS to obtain a solution of N_3-conjugated secondary antibody. To the solution, AdA-DBCO (2.1 mg, 3.5 µmol) was added. After stirring for 6 hours at 25 ºC, the antibody solution was purified by centrifugal filtration (molecular weight cut-off, 30 kDa) with PBS to obtain AdA-conjugated secondary

antibody solution.

Confirmation of AdA conjugation to the secondary goat anti-mouse antibody (IgG)

We confirmed the conjugation of AdA to the secondary antibody using a CB[7]-conjugated bead[1] as follows. The resulting secondary antibody from the above reaction with AdA-DBCO or native secondary antibody (1 μg) was added to a solution of CB[7]-bead (20 μl, swelling volume in PBS) in RIPA buffer (400 μl) in a microtube. The beads in the microtube were gently rotated at 4 degree for 1 hour and washed 3 times with RIPA buffer. After removing RIPA buffer in the tube by gentle aspiration, laemmli sample buffer (Biorad, 2X, 20 μl) with 1,4-dithiothreitol (DTT, 20 mM) and *N*-(1-adamantyl)ethylenediammonium chloride adamantane (TCI, 2 mM) as a strong competitor (2 mM, excess amount to AdA-conjugated antibody) was added to elute the AdA-conjugated antibody from the CB[7]-beads. The recovery of the proteins from the beads (one with AdA-conjugated secondary antibody and the other with native secondary antibody) was confirmed by SDS-PAGE followed by coomassie brilliant blue staining. In the SDS-PAGE (Supplementary Figure 10), a protein band enriched with CB[7]-bead was observed only with the secondary antibody that was reacted with AdA-DBCO as above. It clearly indicated AdA was successfully conjugated to the secondary antibody through the above reaction.

Synthesis of AdA-phenol (Supplementary Figure 3)

4-Hydroxyphenylacetic acid (56 mg, 0.39 mmol), *N,N'*-diisopropylcarbodiimide (DIC, 49 mg, 0.39 mmol) and *N*-hydroxysuccinimide (NHS, 45 mg, 0.39 mmol) were added in a solution of **1**[2] (100 mg, 0.35 mmol) in methylene chloride (MC, 10 ml). Then, trimethylamine was added to the solution in a dropwise manner until it changed to a clear solution and the reaction mixture was stirred for 8 hr at RT. The crude product was purified by silica column chromatography (eluant, MC:MeOH=9:1). The purified compound was dried under a reduced pressure to give **AdA-phenol** (57 mg, 39 %). ^{1}H-NMR (850 MHz, D_2O): δ = 7.19 (d, 2H), 6.87 (d, 2H), 3.69 (br, 2H), 3.62 – 3.60 (br, 6H), 3.50 (s, 2H), 3.38 (t, 2H), 3.14 (t, 2H), 2.16 (br, 3H), 1.84 (br, 6H), 1.73 – 1.71 (br, 3H), 1.64 – 1.61 (br, 3H); ^{13}C NMR (213 MHz, D_2O): δ = 24.8, 28.8, 34.9, 37.9, 38.9, 39.1, 41.6, 57.3, 66.0, 68.7, 69.4, 69.6, 115.8, 126.4, 130.4, 155.3, 175.2, 179.4; HRMS (ESI-MS): m/z calcd. for [M + H^+]: 417.2753, found 417.2754

Synthesis of 4-((2-(2-(2-(((3s,5s,7s)-adamantan-1-yl)amino)ethoxy)ethoxy)ethyl)amino)-4-oxobutanoic acid (AdA-COOH) (Supplementary Figure 4)

Succinic acid (60 mg, 0.50 mmol), DIC (49 mg, 0.39 mmol) and NHS (45 mg, 0.39 mmol) were added in a solution of **1**[2] (90 mg, 0.32 mmol) in MC (10 ml). Then, trimethylamine was added to the solution in a dropwise manner until it changed to a clear solution and the reaction mixture was stirred for 8 hr at RT. The crude product was purified by silica column chromatography (eluant, MC:MeOH=9:1). The purified compound was dried under a reduced pressure to give **AdA-COOH** (70 mg, 57 %). ^{1}H-NMR (850 MHz, CD_3OD): δ = 3.80 (br, 2H), 3.73 (br, 4H), 3.67 (br, 2H), 3.43 (br, 2H), 3.27 (br, 2H), 2.53 (br, 4H), 2.23 (br, 3H), 1.93 (br, 6H), 1.78 – 1.77 (br, 3H), 1.70 – 1.68 (br, 3H); HRMS (ESI-MS): m/z calcd. for [M + H^+]: 383.2546, found 383.2547

Synthesis of AdA-benzylguanine (AdA-BG) (Supplementary Figure 4)

O^6-[4-(Aminomethyl)benzyl]guanine (Matrix Scientific, 44 mg, 0.16 mmol), DIC (20 mg, 0.16 mmol), 1-hydroxybenzotriazole (HOBt, 22 mg, 0.16 mmol) and thrimethylamine (10 μl)

were added in a solution of **AdA-COOH** (30 mg, 0.08 mmol) in dimethylformamide (DMF, 5 ml). Then, was added to the solution and the reaction mixture was stirred for 8 hr at RT. The crude product was purified by silica column chromatography (eluent, MC:MeOH=4:1). The purified compound was dried under a reduced pressure to give **AdA-BG** (22 mg, 44 %). ^{1}H-NMR (850 MHz, D_2O): δ 7.91 (s, 1H), 7.58-7.57 (d, 1H, *J*= 8.2 Hz), 7.36-7.35 (d, 1H, J=7.3 Hz), 5.63 (s, 1H), 4.41 (s, 1H), 3.54-3.52 (m, 6H), 3.49 (m, 2H), 3.31 (m, 2H), 2.77-2.76 (t, 1H, *J*= 5.0), 2.63-2.62 (m, 2H), 2.59-2.58 (m, 2H), 1.96 (br, 3H), 1.58 (br, 6H), 1.56 (br, 3H), 1.49-1.48 (br, 3H); HRMS (ESI-MS): m/z calcd. for [M + H^+]: 635.3669, found 635.3672

Cell culture, transfection and plasmids

COS7 cells were cultured in MEM supplemented with 10% (v/v) fetal bovine serum at 37°C in a humidified CO_2-controlled (5%) incubator. For transfection and transient exprression of proteins, the cells were transfected with plasmids encoding recombinant genes using Lipofectamine 2000 (Invitrogen, Carlsbad, CA). According to the manufacturer's instructions, transfections were performed and the cells were then cultured for additional 24 hr to achieve ectopic expression of the protiens of interest.

Supplementary Figures

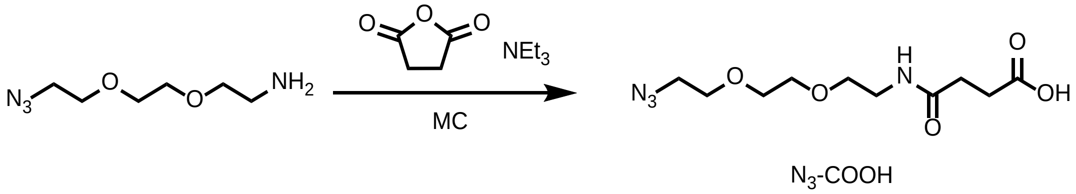

Supplementary Figure 1. Synthesis of N_3-COOH

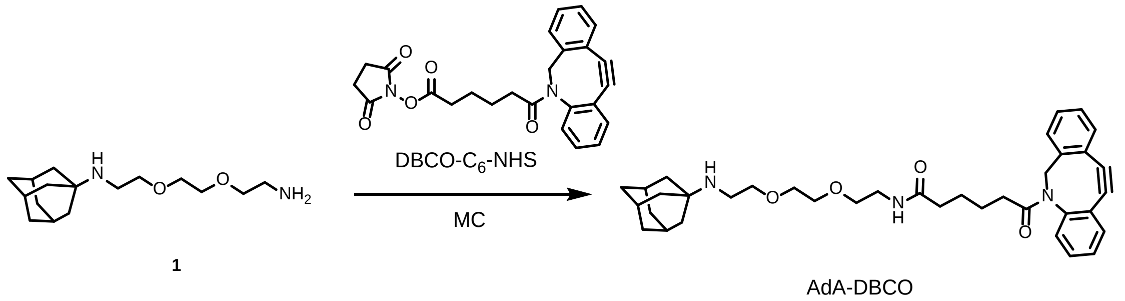

Supplementary Figure 2. Synthesis of AdA-DBCO

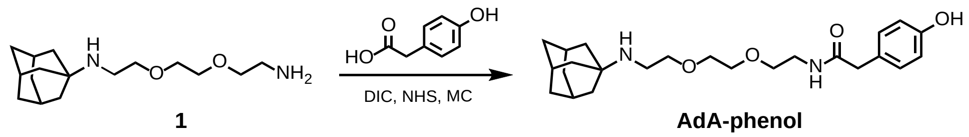

Supplementary Figure 3. Synthesis of AdA-phenol

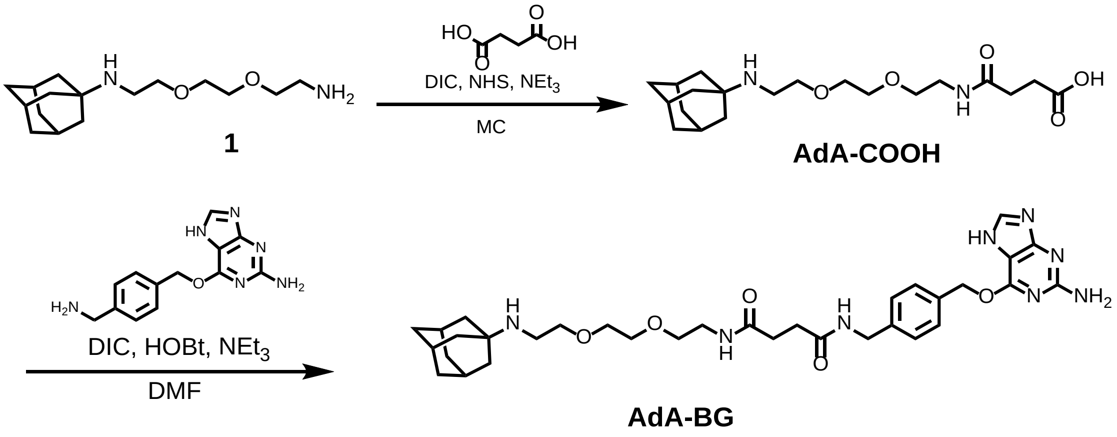

Supplementary Figure 4. Synthesis of AdA-benzylguanine (AdA-BG)

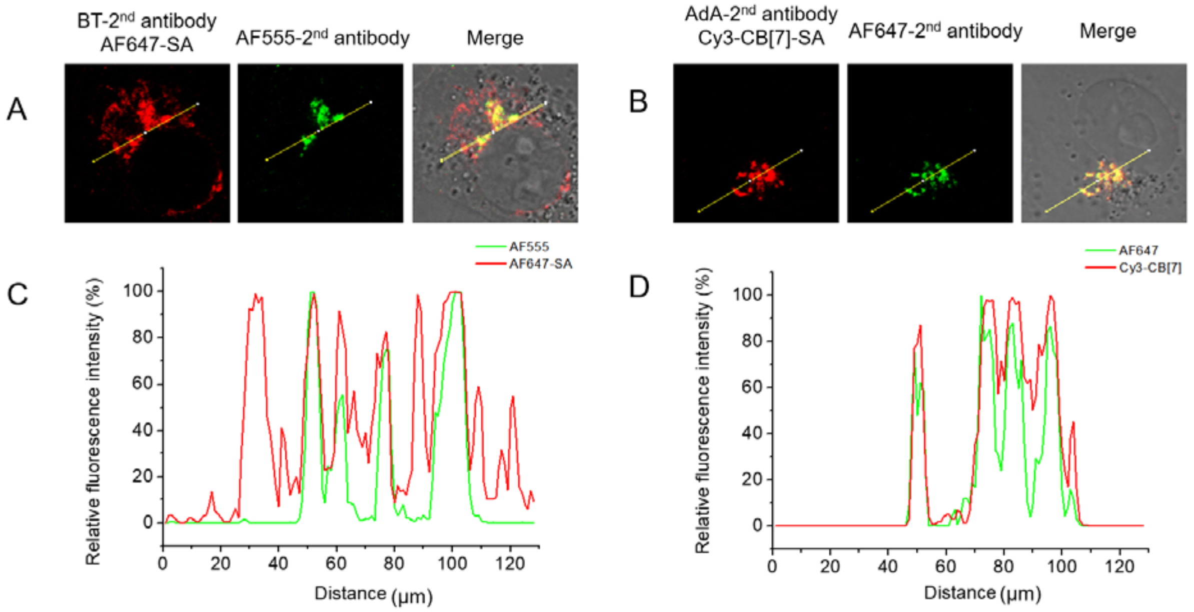

Supplementary Figure 5. A and B) Magnification of cells in Figure 2 in the main text and C and D) thier line profiles for the fluorescence singal from AF647-SA and AF555-secondary antibody, and Cy3-CB[7] and AF646-secondary antibody, respectively.

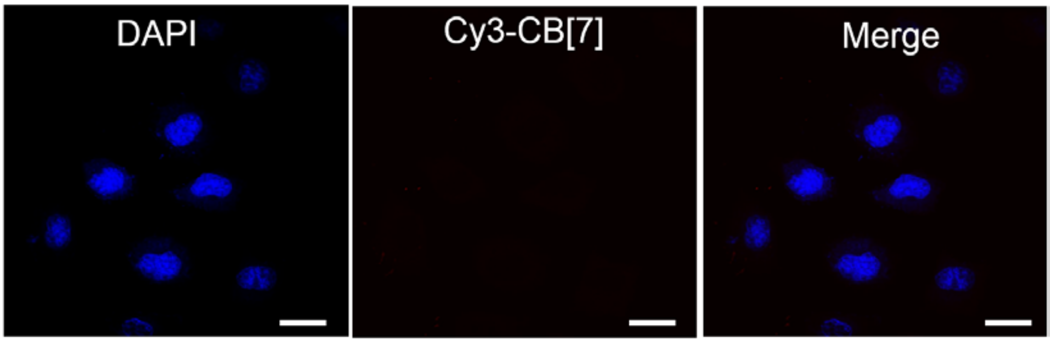

Supplementary Figure 6. Confocal laser scanning microscopy images of COS7 cells (No treated with **FcA-COOH** but with Cy3-CB[7]). Almost no fluorescence signal from Cy3-CB[7] was observed from the cells. Scale bar = 20 μm

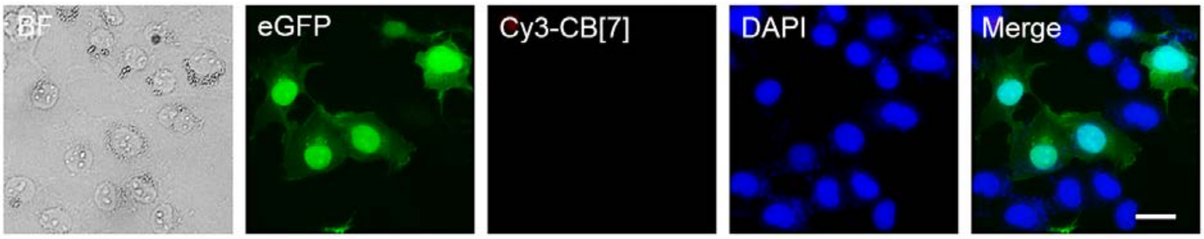

Supplementary Figure 7. Fluorescence microscope images of Cy3-CB[7] treated COS7 cells that have eGFP without FLAG-tag. he cells that expressed eGFP without FLAG-tag were sequentially treated with anti-FLAG primary antibody and HRP-conjugated secondary antibody, then sequentially treated with **AdA-phenol** and H_2O_2, followed by treatment of Cy3-CB[7]. The experiment was performed by following the same procedure as done for Figure 2 in the main text. Scale bar = 20 μm

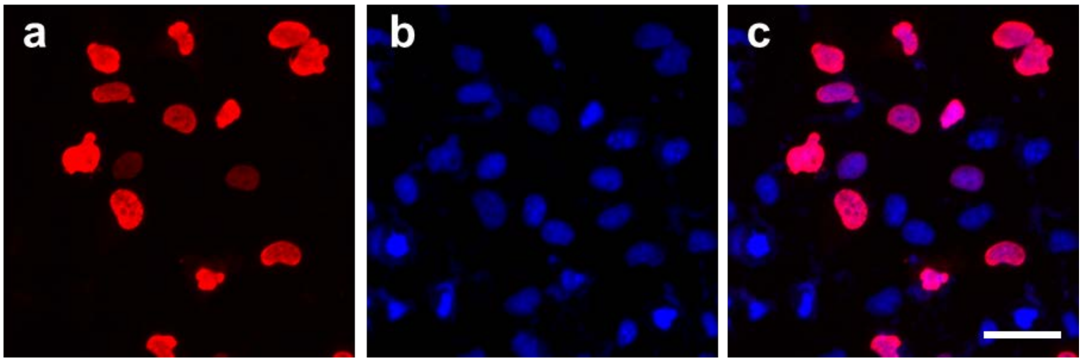

Supplementary Figure 8. Immunofluorescence cell images for FLAG-tag on FLAG-Histone H3 (FLAG-H3). Fluorescence images of (a) Alexa 555, (b) DAPI and (c) merge of (a) and (b) of the fixed cells that was transfected with a plasmid DNA encoding FLAG-H3, after sequantial treamtments of anti-FLAG primary antibody and alexa 555-conjugated anti-Mouse IgG secondary antibody. Scale bar = 50 μm

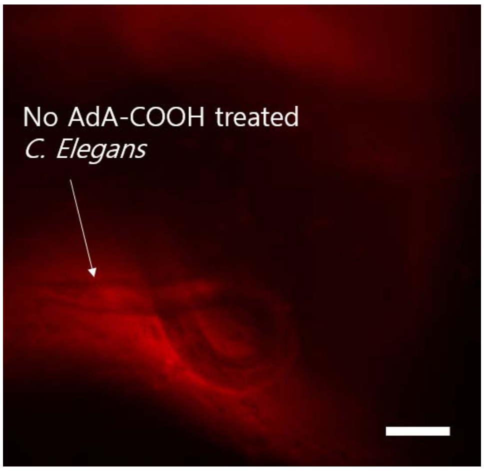

Supplementary Figure 9. A fluorescence microscope image of *C.elegans* (No treated with **AdA-COOH** but with Cy3-CB[7]). The re fluoresence background below the slide glass was used to visualize the *C. elegans* that showed almost no fluorescence signal. Scale bar = 50 μm

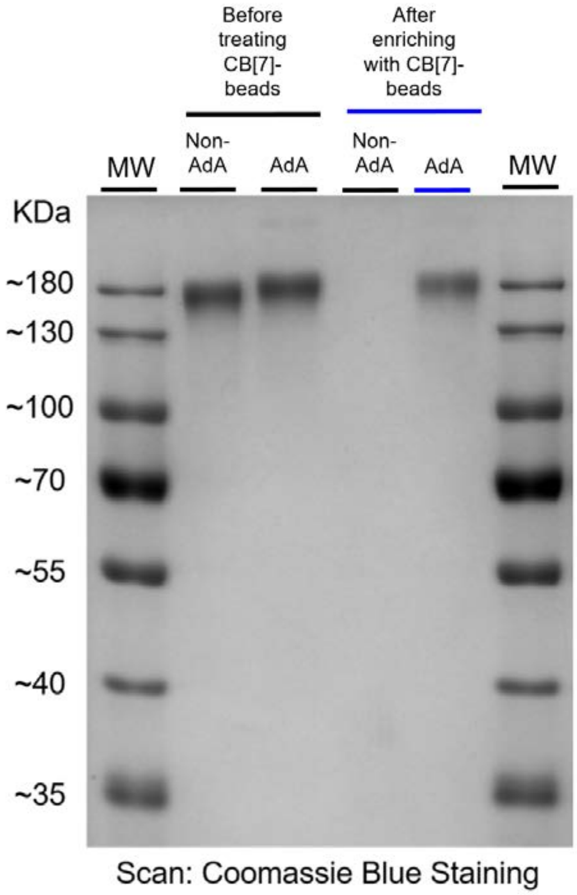

Supplementary Figure 10. SDS-PAGE of AdA-conjugated anti-mouse secondary antibody before/after CB[7]-bead enrichment. AdA-treated antibody or non-treated (native) antibody was incubated with CB[7]-beads. AdA-treated antibody was well-enriched by CB[7]-beads, but the non-treated (native) antibody was not.

Supplementary Tables

Supplementary Table 1. Lists of the plasmids used in this study for the ectopic expression of fusion proteins in mammalian cells.

Name	Features	Promoter/Vector		Details
eGFP		CMV/pEGFP-C1		
FLAG-eGFP	*XbaI*-eGFP-FLAG-Stop-*AgeI*	CMV/pcDNA3.1 myc/his		
FLAGHistone H3	AgeI-Histone H3-FLAG-Stop-*Not1*	CMV/pcDNA3.1 myc/his		
SNAP/eGFP-EGFR	*NotI*-Sp-*AscI*-SNAP-*SacII*-EGFR-*XbaI*-eGFP-Stop-*AgeI*	CMV/pcDNA3.1 myc/his		Sp (signal peptide) for the protein transportation: MRPSGTAGAALLALLA ALCPASRA
mito-V5-APEX2	*NotI*-mito-*BamHI*-V5-APEX-Stop-*XhoI*	CMV/pCDNA3		mito matrix targeting sequence: MLATRVFSLVGKRAISTS VCVRAH<br>V5: GKPIPNPLLGLDST

Supplementary Table 2. The number of fluorescent pixels from Figure 2A-H for calculation of the values for colocalization efficiency (mean ± s.d. %)

Cell	N_{Ov1}	$N_{AF647\text{-}SA}$	N_{Ov2}	$N_{Cy3\text{-}CB[7]}$
1st	1984	5006	3624	3981
2nd	1998	4407	3398	3961
3rd	1459	3046	4190	4567
4th	980	2545	2053	2468
5th	2072	3323	2304	2497
Colocalization efficiency (%)	47 ± 10		89 ± 4	

Supplementary References

1. Lee, D.W. *et al.* Supramolecular fishing for plasma membrane proteins using an ultrastable synthetic host-guest binding pair. *Nat. Chem.* **3**, 154-159 (2011).

2. Gong, B. *et al.* High Affinity Host-Guest FRET Pair for Single-Vesicle Content-Mixing Assay: Observation of Flickering Fusion Events. *J. Am. Chem. Soc.* **137**, 8908-8911 (2015).
